# Supplementary material for: Structure of human TRPM8 channel
Source: Commun Biol. 2023 Oct 19;6:1065. doi: 10.1038/s42003-023-05425-6 (PMC10587237; doi:10.1038/s42003-023-05425-6)
Supplement: Supplementary file 2 — SUPPLEMENTAL MATERIAL [file 42003_2023_5425_MOESM2_ESM.pdf]

Supplementary Information

Structure of human TRPM8 channel

Sergii Palchevskyi[1,2,&], Mariusz Czarnocki-Cieciura[1,&], Giulio Vistoli[3], Silvia Gervasoni[3,4], Elżbieta Nowak[1], Andrea R. Beccari[5], Marcin Nowotny[1,*], Carmine Talarico[5,*]

[&]these authors contributed equally

[*]to whom correspondence should be addressed: carmine.talarico@dompe.com; mnowotny@iimcb.gov.pl

1. Laboratory of Protein Structure, International Institute of Molecular and Cell Biology in Warsaw, 02-109 Warsaw, Poland
2. Cell Signalling Department, Institute of Molecular Biology and Genetics NASU, 03143 Kyiv, Ukraine
3. Dipartimento di Scienze Farmaceutiche, Università degli Studi di Milano, Via Mangiagalli, 25, I-20133 Milano, Italy
4. Department of Physics, University of Cagliari, I-09042 Monserrato, Italy
5. Dompé Farmaceutici SpA, EXSCALATE, Via Tommaso De Amicis, 95, I-80131 Napoli, Italy

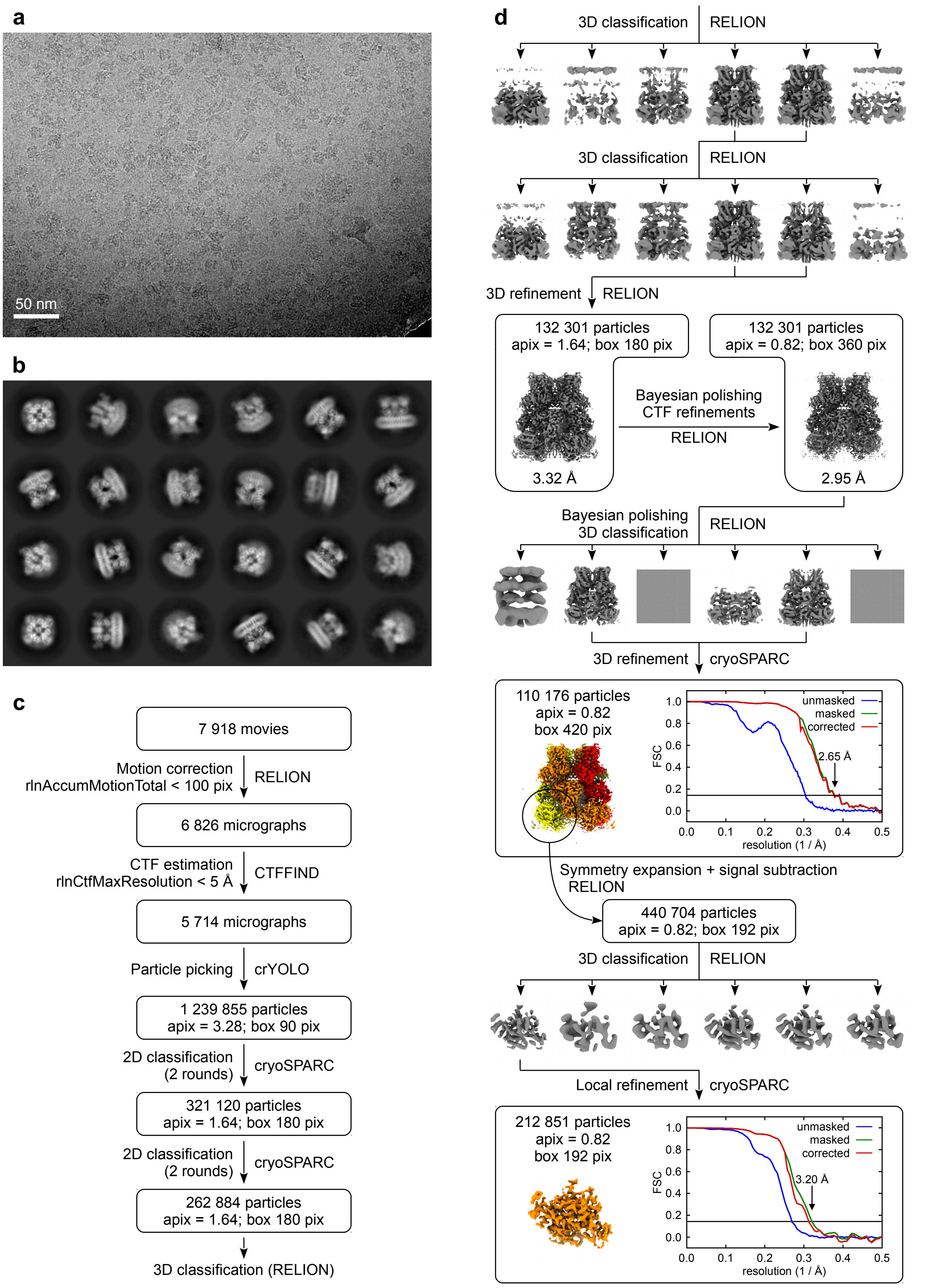

Supplementary Figure 1. Cryo-electron microscopy data processing, related to Figure 1. (**a**) Representative cryo-EM micrograph. (**b**) Selected class averages after last round of two-dimensional classification. (**c**) Initial processing steps and particle curation. (**d**) Three-dimensional reconstruction

pipeline. For the final reconstructions, gold-standard Fourier shell correlation (FSC) curves between two half maps are presented. The horizontal line represents a value of 0.143. Final maps are colored as in Figure 1.

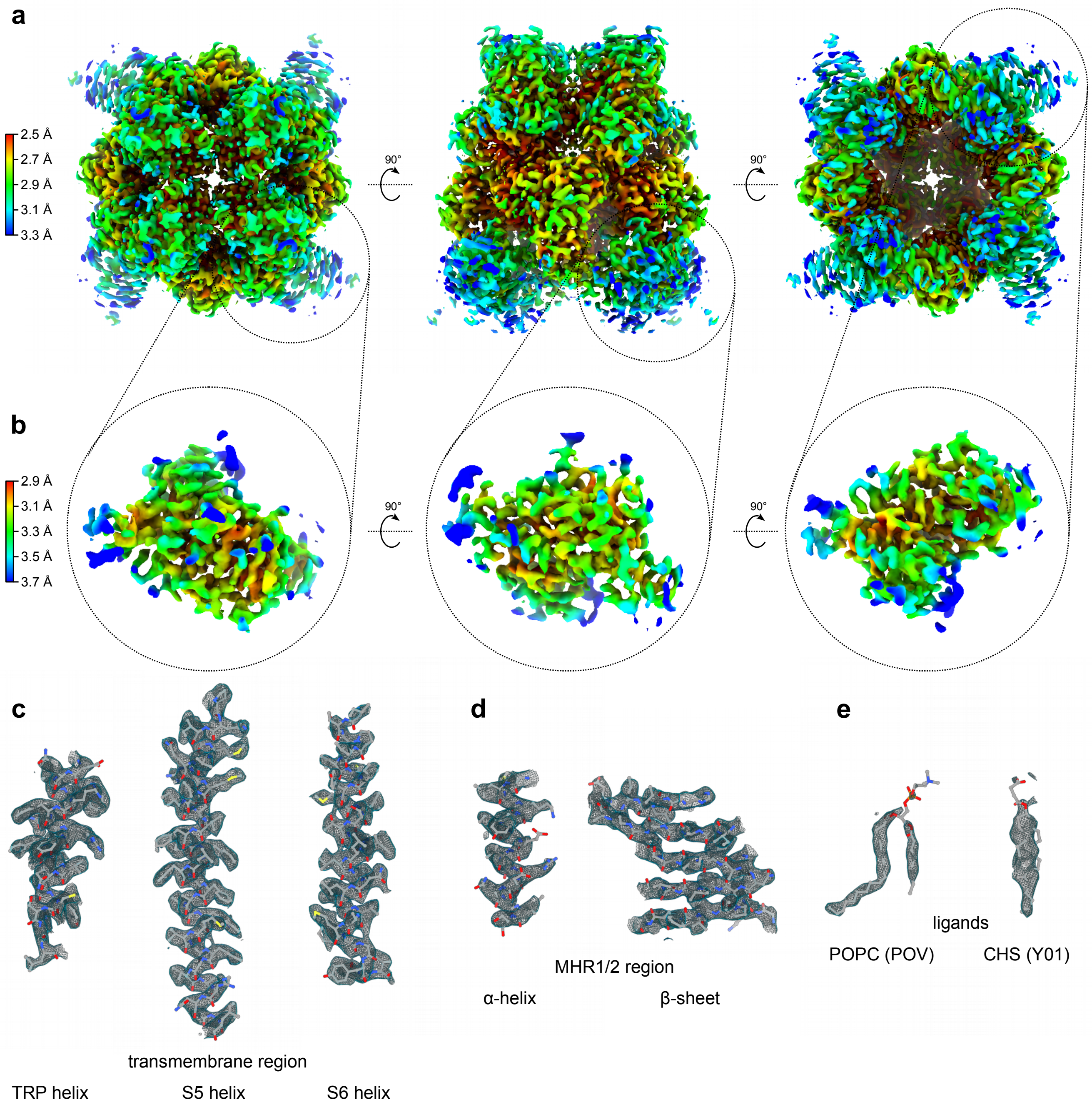

Supplementary Figure 2. Quality of cryo-EM maps, related to Figure 1, Figure 3, and Figure 4. (**a, b**) Local resolution calculated from half maps in cryoSPARC for (a) consensus TRPM8 refinement and (b) focused MHR1/2 refinement. (**c-e**) Quality of the composite cryo-EM map, showing selected secondary structures from the pore region (c) and the MHR1/2 domain (d). (e) Electron microscopy potential density for the selected modeled ligands: phosphatidylcholine (POPC) and cholesteryl hemisuccinate (CHS).

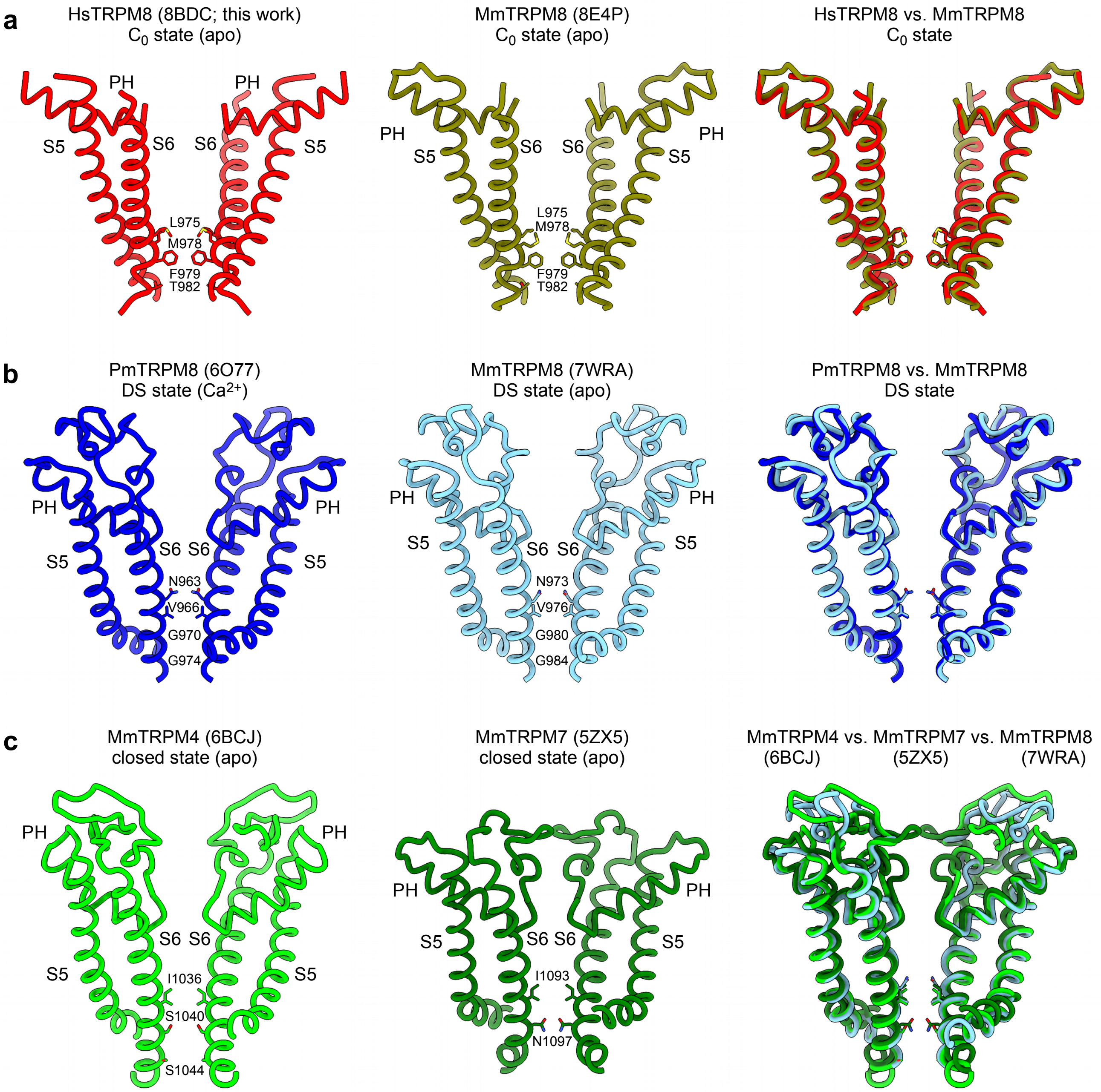

Supplementary Figure 3. Comparison of the pore region of selected TRPM channels, related to Figure 2. (**a**) TRPM8 structures in closed state: HsTRPM8 (PDB ID: 8BDC; this work) and MmTRPM8 (PDB ID: 8E4P). (**b**) MmTRPM8 structure (PDB ID: 7WRA) superimposed with desensitized PmTRPM8 structure (PDB ID: 6O77). (**c**) MmTRPM8 (PDB ID: 7WRA) superimposed with MmTRPM4 (PDB ID: 6BCJ) and MmTRPM7 (PDB ID: 5ZX5). Front and rear subunits were removed for clarity. PH, pore helix.

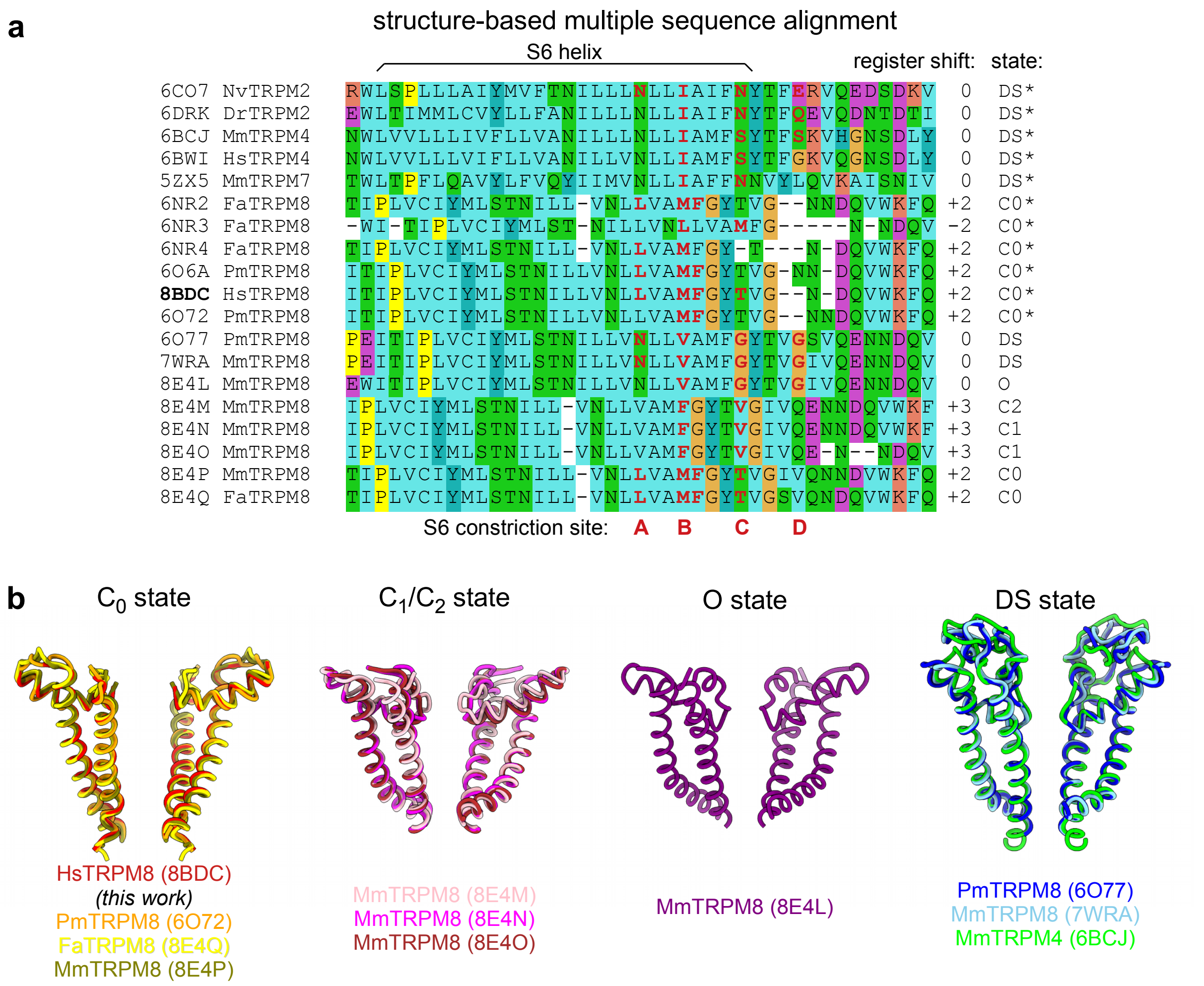

Supplementary Figure 4. Differences in the pore configuration between C_0, C_1/C_2, O, and DS states of TRPM channels, related to Figure 2. (**a**) Structure-based sequence alignment of S6 helices from selected TRPM channels. Residues contributing to constriction site are marked red. Register shift (relative to the reference 6CO7 structure) and established or inferred ('*') state of the channel are indicated. See also Figure 4 in Huffer et al., 2020[1]. (**b**) Comparison of the pore region of selected TRPM channels in indicated states.

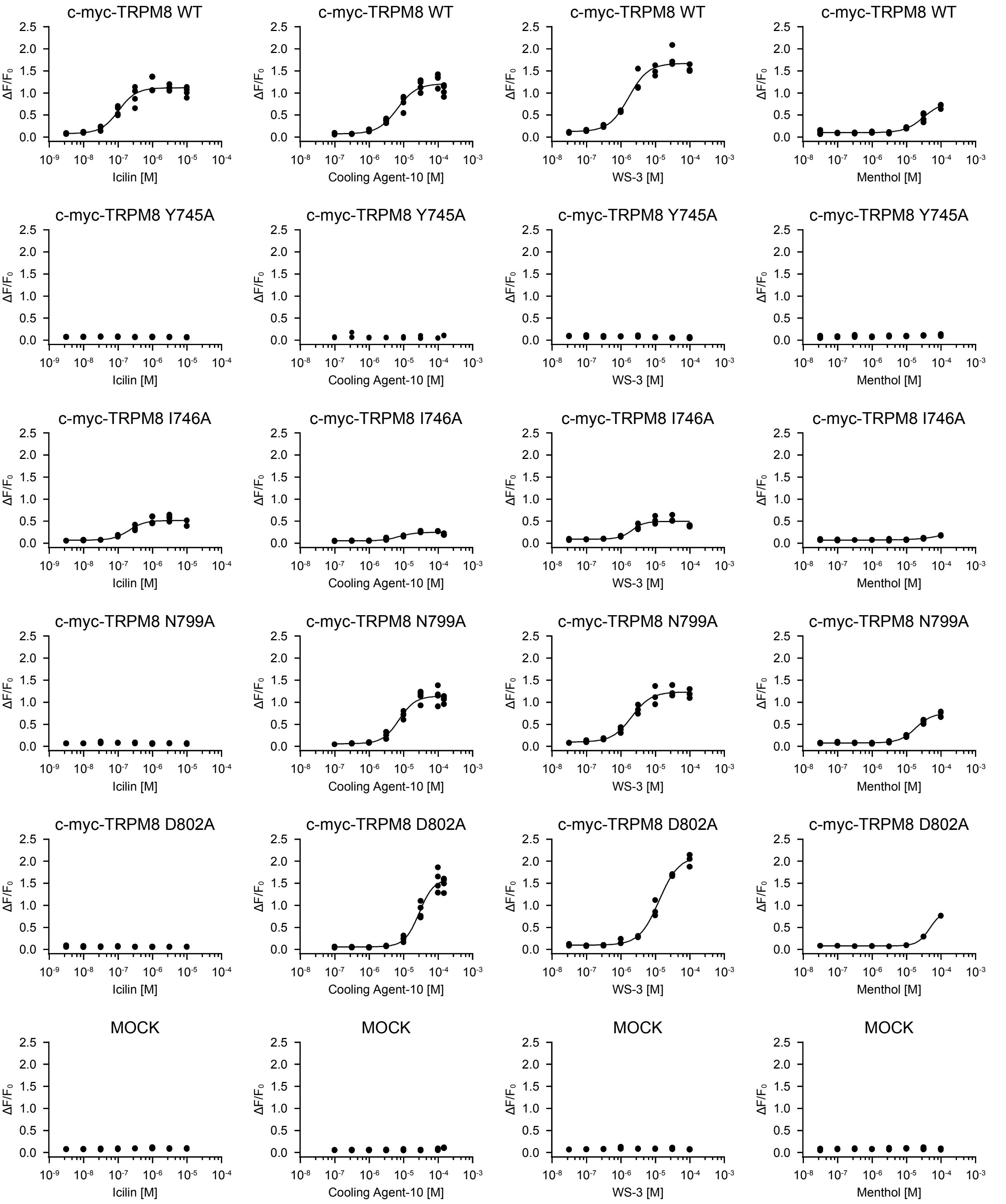

Supplementary Figure 5. HsTRPM8 activation assay, related to Figure 3. Concentration response curves of HsTRPM8 variants activated by selected agonists. Fluorescence signal was normalized to the cellular responses prior to agonist stimulation ($\Delta F/F_0$) and is plotted as individual datapoints along with fitted dose response curves.

Supplementary Table 1. Docking scores for modeling of icilin

	Xscore	ChemPLP	Elec_DD	No. contacts	CHARM6-12
Original pose					
A	-9.05	-59.14	-2.19	6	-41.99
B	-9.05	-58.92	-2.20	6	-41.99
C	-9.05	-86.45	-4.39	11	-44.77
D	-9.23	-72.53	-3.72	11	-40.49
Mean	-9.10	-69.26	-3.12	8.5	-42.31
Rotated pose					
A	-9.23	-49.84	-1.51	8	-40.90
B	-9.42	-81.32	-3.82	7	-40.41
C	-9.31	-60.73	-3.48	8	-40.06
D	-9.17	-80.75	-1.93	7	-38.94
Mean	-9.28	-68.16	-2.68	7.5	-40.08

The XScore and ChemPLP scoring functions encode for overall stability of the generated poses. The Elec_DD score focuses on electrostatic interactions as computed by applying a distance dielectric function. The CHARM6-12 score corresponds to the non-bond Lennard Jones interaction energy using CHARMM parameters. “No. contacts” refers to residues within a 3 Å radius sphere around the ligand. Apart from “No. contacts,” which is dimensionless, all other scores are expressed in kcal/mol.

Supplementary References

1. Huffer, K.E., Aleksandrova, A.A., Jara-Oseguera, A., Forrest, L.R. & Swartz, K.J. Global alignment and assessment of TRP channel transmembrane domain structures to explore functional mechanisms. *Elife* **9** (2020).
