# Supplementary material for: Structure of human TRPM8 channel
Source: Commun Biol. 2023 Oct 19;6:1065. doi: 10.1038/s42003-023-05425-6 (PMC10587237; doi:10.1038/s42003-023-05425-6)
Supplement: Supplementary file 3 — Description of Supplementary Materials [file 42003_2023_5425_MOESM3_ESM.docx]

**Description of Additional Supplementary Files**

**File name:** Supplementary Movie 1

**Description:** Morphing between different conformations of HsTRPM8 resolved by 3D Variability Analysis.
