# Supplementary material for: Structure of human TRPM8 channel
Source: Commun Biol. 2023 Oct 19;6:1065. doi: 10.1038/s42003-023-05425-6 (PMC10587237; doi:10.1038/s42003-023-05425-6)
Supplement: Supplementary file 5 — Reporting Summary [file 42003_2023_5425_MOESM5_ESM.pdf]

nature portfolio

Corresponding author(s): *Double-anonymous peer review submissions: write DAPR and your manuscript number here instead of author names.*

Last updated by author(s): *YYYY-MM-DD*

Reporting Summary

Nature Portfolio wishes to improve the reproducibility of the work that we publish. This form provides structure for consistency and transparency in reporting. For further information on Nature Portfolio policies, see our Editorial Policies and the Editorial Policy Checklist.

Statistics

For all statistical analyses, confirm that the following items are present in the figure legend, table legend, main text, or Methods section.

n/a	Confirmed	
☐	☒	The exact sample size (*n*) for each experimental group/condition, given as a discrete number and unit of measurement
☐	☒	A statement on whether measurements were taken from distinct samples or whether the same sample was measured repeatedly
☒	☐	The statistical test(s) used AND whether they are one- or two-sided<br>*Only common tests should be described solely by name; describe more complex techniques in the Methods section.*
☒	☐	A description of all covariates tested
☒	☐	A description of any assumptions or corrections, such as tests of normality and adjustment for multiple comparisons
☐	☒	A full description of the statistical parameters including central tendency (e.g. means) or other basic estimates (e.g. regression coefficient) AND variation (e.g. standard deviation) or associated estimates of uncertainty (e.g. confidence intervals)
☒	☐	For null hypothesis testing, the test statistic (e.g. *F*, *t*, *r*) with confidence intervals, effect sizes, degrees of freedom and *P* value noted<br>*Give P values as exact values whenever suitable.*
☒	☐	For Bayesian analysis, information on the choice of priors and Markov chain Monte Carlo settings
☒	☐	For hierarchical and complex designs, identification of the appropriate level for tests and full reporting of outcomes
☒	☐	Estimates of effect sizes (e.g. Cohen's *d*, Pearson's *r*), indicating how they were calculated

Our web collection on statistics for biologists contains articles on many of the points above.

Software and code

Policy information about availability of computer code

Data collection	EPU version 2.10.0.5REL
Data analysis	RELION-3.1, cryoSPARC v3.3.2, MotionCor2, CTFFIND-4.1, crYOLO v1.7.4, UCSF Chimera v1.17, UCSF ChimeraX v1.6, AlphaFold v2.1.0, Coot v0.9.8.1, Phenix v1.20.1-4694, VMD-1.9.3, NAMD v2.12, HOLE release 2.2.005, Fr-TM-Align v1.0, VEGA ZZ 3.2.3, GraphPad Prism v6.0.7

For manuscripts utilizing custom algorithms or software that are central to the research but not yet described in published literature, software must be made available to editors and reviewers. We strongly encourage code deposition in a community repository (e.g. GitHub). See the Nature Portfolio guidelines for submitting code & software for further information.

Data

Policy information about availability of data

All manuscripts must include a data availability statement. This statement should provide the following information, where applicable:

- Accession codes, unique identifiers, or web links for publicly available datasets
- A description of any restrictions on data availability
- For clinical datasets or third party data, please ensure that the statement adheres to our policy

The atomic model of HsTRPM8 is available in the Protein Data Bank (PDB) under the accession code 8BDC. The corresponding cryo-EM reconstructions are available in the EM Data Bank under the accession codes EMD-15981 (composite map), EMD-15982 (consensus map), and EMD-15983 (focused pre-MHR + MHR1/2 map).

The models containing icilin molecules and source data for activation assay, multiple sequence alignment, and 3D variability results have been uploaded in public repository on the Zenodo website (DOI: 10.5281/zenodo.8307982).

Research involving human participants, their data, or biological material

Policy information about studies with human participants or human data. See also policy information about sex, gender (identity/presentation), and sexual orientation and race, ethnicity and racism.

Reporting on sex and gender	This study does not involve any human research participants, thus it does not contain any sex- and gender-based analyses.
Reporting on race, ethnicity, or other socially relevant groupings	This study does not involve any human research participants and it does not contain any socially relevant groupings.
Population characteristics	This study does not involve any human research participants and it does not contain any population characteristics.
Recruitment	This study does not involve any human research participants.
Ethics oversight	This study does not involve any human research participants.

Note that full information on the approval of the study protocol must also be provided in the manuscript.

Field-specific reporting

Please select the one below that is the best fit for your research. If you are not sure, read the appropriate sections before making your selection.

☒ Life sciences ☐ Behavioural & social sciences ☐ Ecological, evolutionary & environmental sciences

For a reference copy of the document with all sections, see nature.com/documents/nr-reporting-summary-flat.pdf

Life sciences study design

All studies must disclose on these points even when the disclosure is negative.

Sample size	HsTRPM8 activation assays were performed in triplicate or tetraplicate which allowed for unambiguous fitting of dose response curves.
Data exclusions	For HsTRPM8 activation assay no data were excluded from preliminary fitting. Dose response curves and EC50 values are reported only for unambiguous fits.
Replication	We confirm that all the attempts at replication were successful.
Randomization	This study does not contain experimental groups other than protein mutants.
Blinding	This study does not contain experimental groups other than protein mutants.

Reporting for specific materials, systems and methods

We require information from authors about some types of materials, experimental systems and methods used in many studies. Here, indicate whether each material, system or method listed is relevant to your study. If you are not sure if a list item applies to your research, read the appropriate section before selecting a response.

Materials & experimental systems

n/a	Involved in the study	
☒	☐	Antibodies
☐	☒	Eukaryotic cell lines
☒	☐	Palaeontology and archaeology
☒	☐	Animals and other organisms
☒	☐	Clinical data
☒	☐	Dual use research of concern
☒	☐	Plants

Methods

n/a	Involved in the study	
☒	☐	ChIP-seq
☒	☐	Flow cytometry
☒	☐	MRI-based neuroimaging

Eukaryotic cell lines

Policy information about cell lines and Sex and Gender in Research

Cell line source(s)	Sf9 insect cell line (commercial source); HEK293 cell line (commercial source)
Authentication	Sf9 and HEK293 cell lines were not authenticated - are derived from commercial source;
Mycoplasma contamination	All cell lines were routinely tested for mycoplasma contamination (with negative results)
Commonly misidentified lines (See ICLAC register)	Not applicable for HEK93 and Sf9 insect cell lines from commercial source
